# Circadian monitoring as an aging predictor

**DOI:** 10.1038/s41598-018-33195-3

**Published:** 2018-10-09

**Authors:** A. Martinez-Nicolas, J. A. Madrid, F. J. García, M. Campos, M. T. Moreno-Casbas, P. F. Almaida-Pagán, A. Lucas-Sánchez, M. A. Rol

**Affiliations:** 1 Chronobiology Lab, Department of Physiology, College of Biology, University of Murcia, Mare Nostrum Campus, IUIE, IMIB-Arrixaca, Murcia, Spain; 2 Ciber Fragilidad y Envejecimiento Saludable (CIBERFES), Madrid, Spain; 3 Geriatrics Section, Hospital Virgen del Valle, Toledo, Spain; 4 Department of Computer Science and Systems, University of Murcia, IMIB-Arrixaca, Murcia, Spain; 5 Nursing and Healthcare Research Unit (Investén-isciii), Madrid, Spain

## Abstract

The ageing process is associated with sleep and circadian rhythm (SCR) frailty, as well as greater sensitivity to chronodisruption. This is essentially due to reduced day/night contrast, decreased sensitivity to light, napping and a more sedentary lifestyle. Thus, the aim of this study is to develop an algorithm to identify a SCR phenotype as belonging to young or aged subjects. To do this, 44 young and 44 aged subjects were recruited, and their distal skin temperature (DST), activity, body position, light, environmental temperature and the integrated variable TAP rhythms were recorded under free-living conditions for five consecutive workdays. Each variable yielded an individual decision tree to differentiate between young and elderly subjects (DST, activity, position, light, environmental temperature and TAP), with agreement rates of between 76.1% (light) and 92% (TAP). These decision trees were combined into a unique decision tree that reached an agreement rate of 95.3% (4 errors out of 88, all of them around the cut-off point). Age-related SCR changes were very significant, thus allowing to discriminate accurately between young and aged people when implemented in decision trees. This is useful to identify chronodisrupted populations that could benefit from chronoenhancement strategies.

## Introduction

The circadian system is composed of a hierarchically organized set of structures which are responsible for generating circadian rhythms and synchronizing them to the environmental conditions. This system includes inputs, the central pacemaker, outputs, a series of peripheral oscillators and the connections among them^1^. The central pacemaker is located in the suprachiasmatic nuclei of the hypothalamus (SCN), which sends temporal information to the entire organism through humoral and nervous signals. It is reset everyday by light input from cones, rods and intrinsically photosensitive retinal ganglion cells (ipRGCs)^2,3^.

As occurs for almost every physiological function, the human circadian system progressively loses functionality with age^4^. The aging process impairs circadian system inputs mainly through pupillary myosis, reduced transmission of blue light by the crystalline lens^5^, a reduction in the density of ipRGCs in the retina and the atrophy of ipRGCs dendritic trees^6^. The SCN itself is also susceptible to age-related impairment in the form of neuronal degeneration^7^ and a lower release of AVP and VIP neurotransmitters^8^. In addition, studies on animal models have shown a decrease in neural coupling^8^, a reduction of GABAergic synapses^9^ and decreased neuronal firing amplitude^10^ in aged animals. Finally, aging also alters outputs, which causes a morning preference over evening preference^11^. Furthermore, overt rhythms usually display lower amplitudes, greater fragmentation and a phase advance^7,8,12–14^.

With regard to sleep, aged people tend to wake up earlier and experience more fragmented sleep and daytime sleepiness than other adults. They show higher interdaily stability^15,16^ and higher activity values during night-time hours, which is associated with lower sleep efficiency^17^. They also expose themselves to higher light intensities throughout the day^18^. Furthermore, thermoregulation is also impaired due to a reduction in the efficacy of heat retention, and to heat loss caused by impaired cutaneous vasoconstriction and vasodilatation mechanisms, together with a reduction in thermal sensitivity, which is more pronounced with warm temperatures^19^. The results of these thermoregulatory changes are a reduction in mean core body temperature, a phase advance and a decrease in amplitude of the core body temperature rhythm^19,20^, whereas distal skin temperature (DST) presents higher mean values, especially during the daytime (related to daytime sleepiness^21^), and a phase advance^22^.

In summary, aged individuals become more sensitive to chronodisruption and sleep and circadian rhythm (SCR) frailty^23^, essentially due to a lack of day/night contrast^24^, reduced sensitivity to light^5,6^, napping^25^ and a more sedentary lifestyle^17^.

Taking into account that at least part of these negative effects on circadian health could be reduced by prompting changes in the habits of the elderly, the aim of this study is to develop an algorithm to identify SCR frailty.

## Results

### Daily patterns

**Figure 1:**
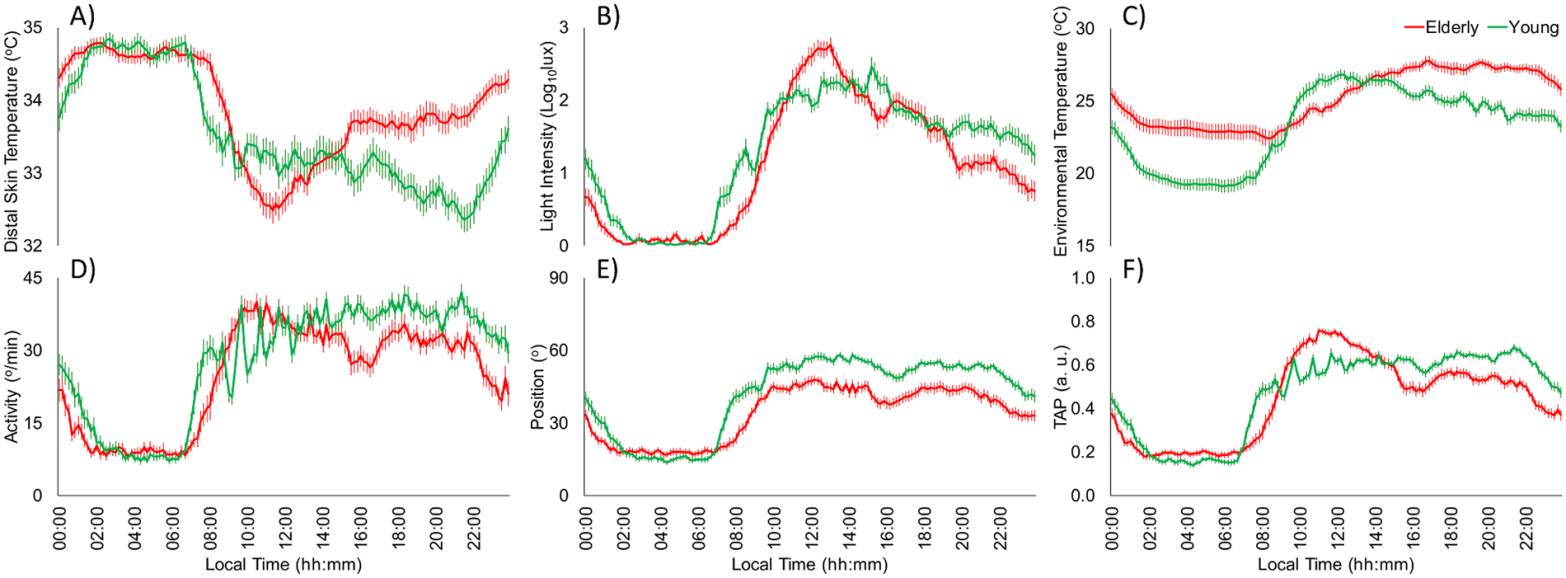
Mean-waveforms for young (green line, n: 44) and aged participants (red line, n: 44) for: (**A**) distal skin temperature, (**B**) light exposure, (**C**) environmental temperature, (**D**) activity, (**E**) body position and (**F**) the integrated variable TAP. All variables are expressed as the mean ± SEM.

Daily mean patterns of all recorded variables for young (n: 44) and aged participants (n: 44) are shown in Fig. 1, while their rhythmic characteristics are detailed in Table 1. The distal skin temperature (DST) pattern of young and aged participants (Fig. 1A) showed common rhythmic characteristics, with high and relatively stable values during sleep time (from 00:10 to 08:00 h) and low and highly variable values during the active phase (from 08:10 to 00:00 h). However, in young participants, daytime is characterized by a decrease that bottoms out in the late evening (from 20:00 to 22:00 h), corresponding to the “wake maintenance zone”, while in aged participants, the lowest daytime values appeared in the morning (from 10:00 to 12:00 h), increasing until they reached a plateau (33.6 °C) from 16:00 to 22:00 h. Finally, aged participants showed higher DST mean values (Mean), stability (IS) and robustness (CFI), as well as a phase advance of TMIN, as shown in Table 1.

**Table 1:** Non-parametrical indexes of circadian rhythms according to age group (elderly participants, n = 44; young participants, n = 44).

		IS	IV	RA	MIN	MAX	TMIN	TMAX	CFI	MEAN
DST	Y	0.57 ± 0.02	0.20 ± 0.02	0.03 ± 0.00	32.76 ± 0.15	34.87 ± 0.08	16:25 ± 00:39	02:49 ± 00:35	0.50 ± 0.01	33.57 ± 0.09
	E	0.69 ± 0.02^‡^	0.20 ± 0.01	0.03 ± 0.00	33.14 ± 0.12	34.85 ± 0.07	13:50 ± 00:18^‡^	03:03 ± 00:27	0.54 ± 0.01^#^	33.92 ± 0.09^#^
L	Y	0.70 ± 0.02	0.25 ± 0.01	0.98 ± 0.01	0.03 ± 0.01	2.08 ± 0.06	04:25 ± 00:10	15:22 ± 00:21	0.85 ± 0.01	1.27 ± 0.04
	E	0.76 ± 0.02	0.28 ± 0.02	0.97 ± 0.01	0.03 ± 0.01	2.05 ± 0.06	03:52 ± 00:14	14:46 ± 00:14	0.86 ± 0.01	1.08 ± 0.04^#^
ET	Y	0.63 ± 0.03	0.10 ± 0.01	0.16 ± 0.01	19.00 ± 0.35	26.18 ± 0.24	05:08 ± 00:16	15:30 ± 00:25	0.58 ± 0.01	23.19 ± 0.25
	E	0.71 ± 0.02^*^	0.09 ± 0.00	0.11 ± 0.00^‡^	22.15 ± 0.35^‡^	27.63 ± 0.32^#^	06:22 ± 00:31	17:32 ± 00:36^#^	0.59 ± 0.01	25.15 ± 0.32^‡^
ACT	Y	0.59 ± 0.01	0.71 ± 0.02	0.68 ± 0.01	7.52 ± 0.35	39.02 ± 0.76	04:11 ± 00:10	17:22 ± 00:20	0.64 ± 0.01	27.64 ± 0.56
	E	0.54 ± 0.01^#^	0.72 ± 0.03	0.62 ± 0.02^*^	7.98 ± 0.49	34.34 ± 0.90^‡^	03:51 ± 00:13	15:08 ± 00:20^‡^	0.60 ± 0.01^*^	24.62 ± 0.65^‡^
POS	Y	0.70 ± 0.02	0.26 ± 0.01	0.59 ± 0.02	14.33 ± 0.72	55.40 ± 0.83	04:14 ± 00:12	15:22 ± 00:17	0.72 ± 0.01	41.04 ± 0.62
	E	0.60 ± 0.02^#^	0.34 ± 0.02^‡^	0.49 ± 0.02^‡^	15.84 ± 1.00	44.82 ± 1.38^‡^	03:20 ± 00:32	14:52 ± 00:25	0.64 ± 0.02^‡^	34.00 ± 0.99^‡^
TAP	Y	0.74 ± 0.02	0.25 ± 0.01	0.62 ± 0.02	0.15 ± 0.01	0.64 ± 0.01	04:15 ± 00:10	17:07 ± 00:18	0.74 ± 0.01	0.47 ± 0.00
	E	0.78 ± 0.02	0.31 ± 0.02^‡^	0.56 ± 0.02	0.18 ± 0.01^*^	0.63 ± 0.01	03:47 ± 00:14	14:15 ± 00:09^‡^	0.73 ± 0.01	0.43 ± 0.00^‡^

Main characteristics of the circadian rhythms and environmental preferences studied (distal skin temperature, DST; light exposure, L; environmental temperature, ET; activity, ACT, body position, POS and integrated variable TAP, TAP) for young and elderly participants (Y and E, respectively). Interdaily stability (IS), intradaily variability (IV), relative amplitude (RA), mean of the 10 consecutive hours with the lowest values (MIN), and the 5 consecutive hours with the highest values (MAX) for distal skin temperature and their respective timing (TMIN and TMAX), 5 consecutive hours with the lowest values (MIN), and the 10 consecutive hours with the highest values (MAX) for light exposure, environmental temperature, activity, body position and the integrated variable TAP and their respective timing (TMIN and TMAX), circadian function index (CFI) and mean value (MEAN). IS, IV, RA and CFI have no units, MIN, MAX and MEAN are expressed in the units of their respective variable (DST in °C, L in log_10_lux, ET in °C, ACT in °/min, POS in degrees and TAP in arbitrary units) while TMIN and TMAX are expressed in hours (hh:mm). Values are expressed as the mean ± SEM. ^‡^p < 0.001, ^#^p < 0.01 and *p < 0.05, according to General Linear Model controlling for gender with *post-hoc* Benjamini-Hochberg procedure for multiple comparisons.

The mean light exposure pattern (Fig. 1B) exhibited low values (lower than 10 lux) at night and higher values during daytime, as expected. Young participants were exposed to less than 10 lux from 00:30 to 08:00 h, and experienced a gradual increase to a maximum level at 15:00 h, followed by a slow decrease. However, aged participants were exposed to less than 10 lux from 22:30 to 09:00 h, reaching the maximum level at 13:00 h; this was then followed by a pronounced and continuous decrease from that moment on. With regard to the non-parametrical indexes (see Table 1), aged participants were exposed to a lower mean light intensity (Mean). According to data in Table 2, elder were exposed longer to darkness throughout the day (<10 lux; 13:16 ± 00:30 *vs*. 10:35 ± 00:27 h respectively, p < 0.001) and also to the highest light intensity (>10000 lux; 00:33 ± 00:04 *vs*. 00:18 ± 00:03 h respectively, p < 0.01) while they spent less time at intermediate light intensities (100–1000 lux; 04:35 ± 00:20 *vs*. 06:32 ± 00:23 h respectively, p < 0.01) than young participants. Finally, time exposed to 10–100 lux and more than 1000 lux categories (1000–10000 and >10000 lux) did not differ between both age groups among morning, evening and night period.

**Table 2:** Time of exposure to different light intensities by day period and age group (elderly participants, n = 44; young participants, n = 44).

Group	Period	<10 lux	10–100 lux	100–1000 lux	1000–10000 lux	>10000 lux
Young	Morning	01:20 ± 00:10^a^	02:23 ± 00:08^b^	03:15 ± 00:12^c^	00:45 ± 00:06^d^	00:18 ± 00:03^d^
	Evening	02:01 ± 00:11^a†^	02:30 ± 00:10^ab^	02:53 ± 00:14^b^	00:34 ± 00:07^c^	00:02 ± 00:07^c^
	Night	07:00 ± 00:12^a†‡^	00:23 ± 00:04^bc†‡^	00:31 ± 00:04^b†‡^	00:05 ± 00:02 ^cd†‡^	00:00 ± 00:00^d^
Elderly	Morning	02:17 ± 00:11^a*^	01:56 ± 00:09^a^	02:20 ± 00:08^a*^	00:59 ± 00:04^b^	00:29 ± 00:04^b^
	Evening	03:18 ± 00:16^a†*^	02:05 ± 00:10^b^	02:06 ± 00:14^b*^	00:25 ± 00:04^c†^	00:06 ± 00:02^c^
	Night	07:30 ± 00:11^a†‡*^	00:14 ± 00:03^b†‡^	00:14 ± 00:02^b†‡*^	00:03 ± 00:01^b†^	00:00 ± 00:00^b†^

Time spent at different light intensities during three time intervals during daytime (morning from 08:00 to 15:50, evening from 16:00 to 23:50 and night from 00:00 to 07:50) for young and elderly participants. All the data are expressed as mean ± SEM (hh:mm). *Indicates statistically significant differences with the young group in the same light intensity and period, ^†^Indicates statistically significant differences with morning in the same light intensity and age group, ^‡^Indicates statistically significant differences with evening in the same light intensity and age group and different letters indicates statistically significant differences among light intensities in the same time window and age group according to General Linear Model repeated measures controlling for gender with *post-hoc* Benjamini-Hochberg procedure for multiple comparisons.

The environmental temperature rhythm (Fig. 1C) exhibited low values at night and high values during the daytime. Young participants were exposed to temperatures of around 20 °C at night (from 01:00 to 08:00 h) and around 25 °C from 10:00 to 00:00 h. However, aged participants were exposed to temperatures of around 23 °C from 01:00 to 09:00 h, reaching 27 °C during the second half of the day (from 15:00 to 23:00 h). In addition, as shown in Table 1, aged participants were exposed to a lower amplitude (RA), more stable (IS) and phase-delayed (TMAX) environmental temperature pattern, with higher mean temperatures throughout the day (MIN, MAX and Mean).

As expected, activity and body position (Fig. 1D,E) exhibited stable, low values at night and higher and more variable values during the active phase, showing a slight decrease that coincides with the Spanish postprandial period (from 15:00 to 17:00 h), which was not characteristic of the activity in young participants. Globally, activity and body position were lower in aged than in young participants, especially during daytime, whereas at night, the values of aged participants tended to be higher than those of the young subjects. The non-parametrical indexes (Table 1) resulted in a lower robustness (CFI), stability (IS), amplitude (RA) and daytime (MAX) and mean values (Mean) for activity and body position patterns in aged participants as compared to young subjects. In addition, the body position pattern was more fragmented and the active phase for activity was advanced (TMAX) in the elders.

The integrated variable TAP (Fig. 1F) showed low and stable values at night, and high and variable values during the daytime. Young participants showed a plateau from around 10:00 to 22:00 h, and then exhibited a fast decrease towards the rest period. Aged participants, however, reached maximum TAP values around midday. Finally, aged participants showed higher fragmentation (IV) for TAP patterns than young participants (see Table 1), along with a phase advance in maximum values (TMAX), lower mean values (Mean) and higher values during the night (MIN).

### Synchronization to sleep offset

Sleep parameters from the diaries are shown in Table 3. Aged participants went to bed earlier (sleep onset) and got up later (sleep offset) than young participants; they thus spent longer time in bed without any differences in midsleep.

**Table 3:** Sleep parameters by age group (elderly participants, n = 44; young participants, n = 44).

	Young	Elderly
Sleep onset	00:57 ± 00:08	23:55 ± 00:10^‡^
Sleep offset	07:45 ± 00:07	08:39 ± 00:07^‡^
Sleep length	06:47 ± 00:09	08:44 ± 00:10^‡^
Midsleep	04:21 ± 00:06	04:17 ± 00:07

Values are expressed as the mean ± SEM (hh:mm). ^‡^p < 0.001, according to the General Linear Model controlled by gender.

**Figure 2:**
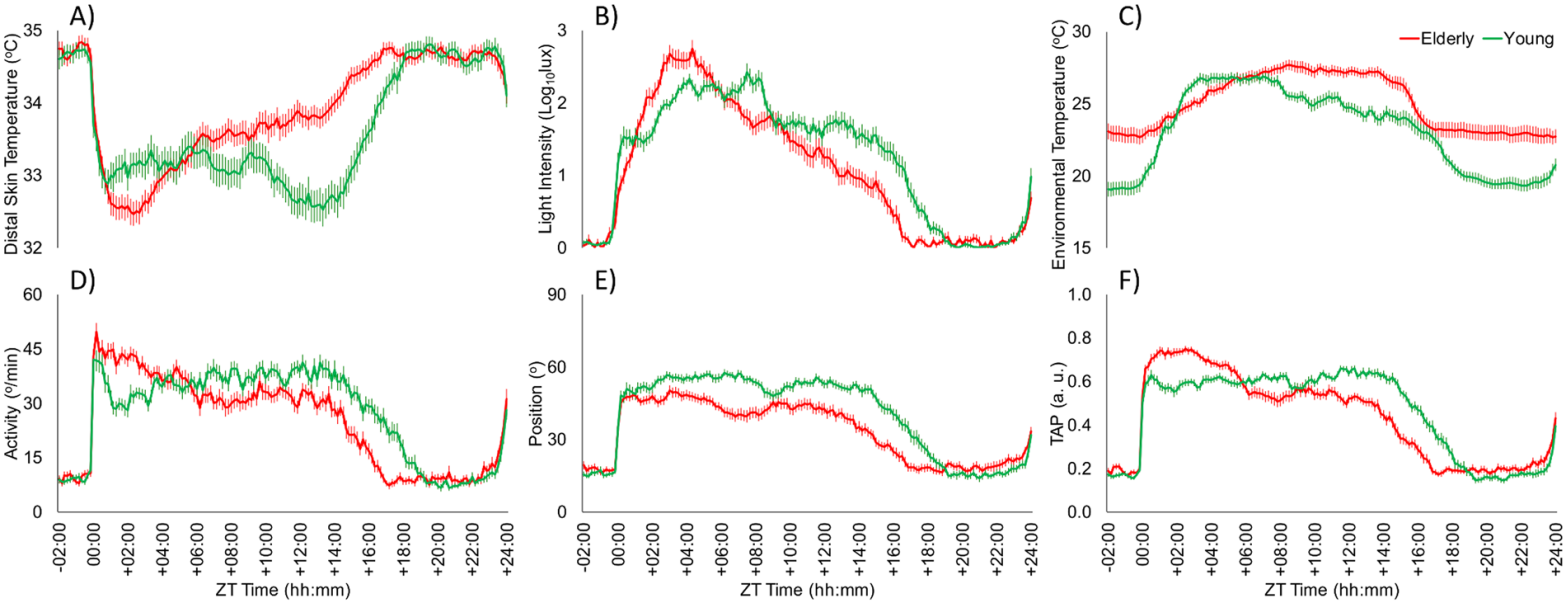
Mean-waveforms standardized for sleep offset for young (green line, n: 44) and aged participants (red line, n: 44) for: (**A**) distal skin temperature, (**B**) light exposure, (**C**) environmental temperature, (**D**) activity, (**E**) body position and the (**F**) the integrated variable TAP. All variables are expressed as the mean ± SEM.

Daily mean patterns of the recorded variables standardized by sleep offset for young (n: 44) and aged participants (n: 44) are shown in Fig. 2. These figures highlight two important aspects: (1) the generalized phase advance in the aged participants as compared to young subjects (see Supplementary Material 1 for the statistical analysis); (2) the active phase shortening (and the lengthening of the rest phase) for aged participants.

### Decision trees

**Figure 3:**
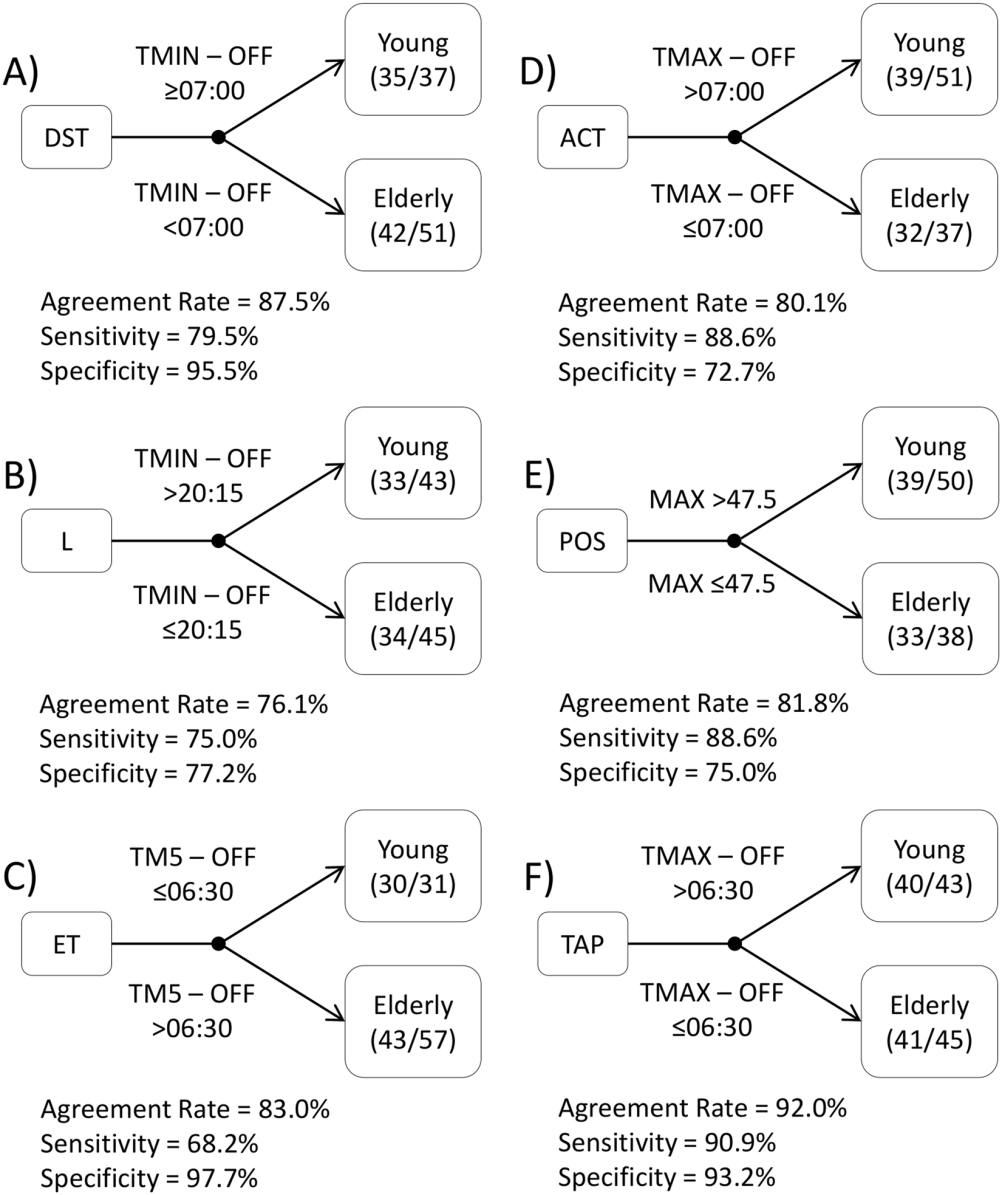
Individual variable decision trees. Decision trees for classifying subjects as young or aged based on distal skin temperature (DST) by TMIN-OFF (**A**), light exposure (L) by TMIN-OFF (**B**), environmental temperature (ET) by TM5-OFF (**C**), activity (ACT) by TMAX-OFF (**D**), body position (POS) by the M10 value (**E**) and the integrated variable TAP by TMAX-OFF (**F**). Agreement rate, sensitivity (test’s ability to correctly detect young subjects) and specificity (test’s ability to correctly detect elderly subjects) are indicated below each variable’s name, classification rules appear immediately to the right (upper part for young, and bottom part for aged participants), and end nodes are included on the right, indicating the correct classifications with respect to the total number of individuals in each particular group.

The decision trees performed for every variable are shown in Fig. 3. The DST decision tree (Fig. 3A) reached an agreement rate of 87.5% (Sensitivity = 79.5%; Specificity = 95.5%), using TMIN at 07:00 hours after sleep offset as the classification rule. For light exposure (Fig. 3B), the classification rule was whether TMIN occurred earlier or later than 20:15 hours after sleep offset, which yielded a 76.1% agreement rate (Sensitivity = 75.0%; Specificity = 77.2%). The environmental temperature decision tree classification rule was TM5-OFF occurring at 06:30 hours (Fig. 3C), which reached an 83.0% agreement rate (Sensitivity = 68.2%; Specificity = 97.7%). The decision tree for activity yielded an agreement rate of 80.1% (Fig. 3D), using TMAX earlier or later than 07:00 hours after sleep offset as the classification rule (Sensitivity = 88.6%; Specificity = 72.7%). For position pattern (Fig. 3E), the classification rule was 47.5° as MAX, with a 91.8% agreement rate (Sensitivity = 88.6%; Specificity = 75.0%). Finally, the TAP variable decision tree yielded an agreement rate of 92.0% (Sensitivity = 90.9%; Specificity = 93.2%), using a TMAX occurring at 06:30 hours after sleep offset (Fig. 3F) for discrimination.

**Figure 4:**
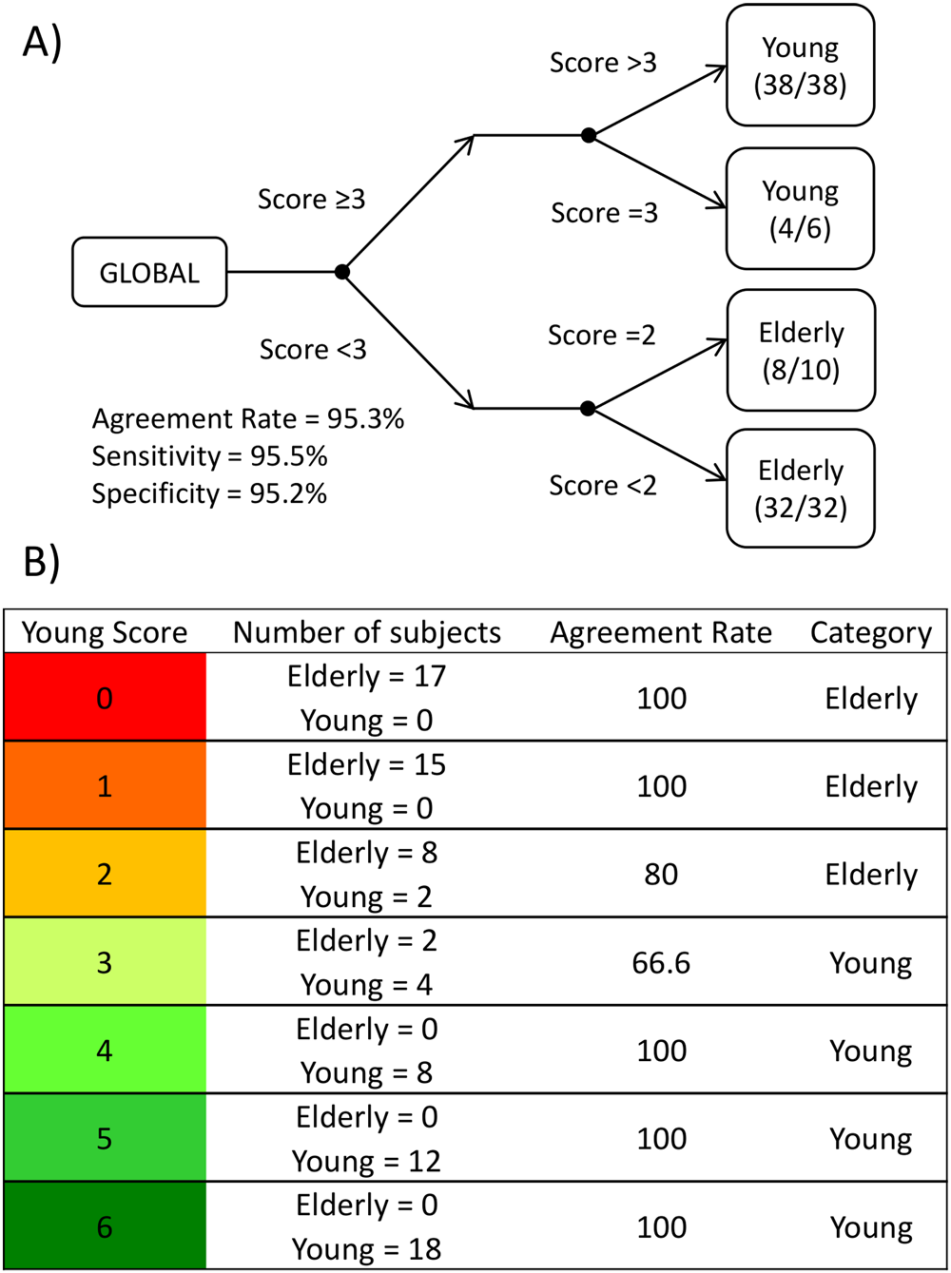
Global decision tree. Section A shows a decision tree that integrates individual variables. Each participant scores 1 point if he/she is classified as young in the decision trees for one variable (distal skin temperature, light exposure, environmental temperature, activity, body position and the integrated variable TAP) and 0 when he/she is classified as aged. The global rate of agreement, sensitivity (test’s ability to correctly detect young subjects) and specificity (test’s ability to correctly detect elderly subjects) are shown below the start of the tree (GLOBAL). When a subject scores three or more points, he/she is classified as young; otherwise he/she is denoted as aged. Section B shows the agreement rate, classification category and number of subjects for each age group and score.

**Figure 5:**
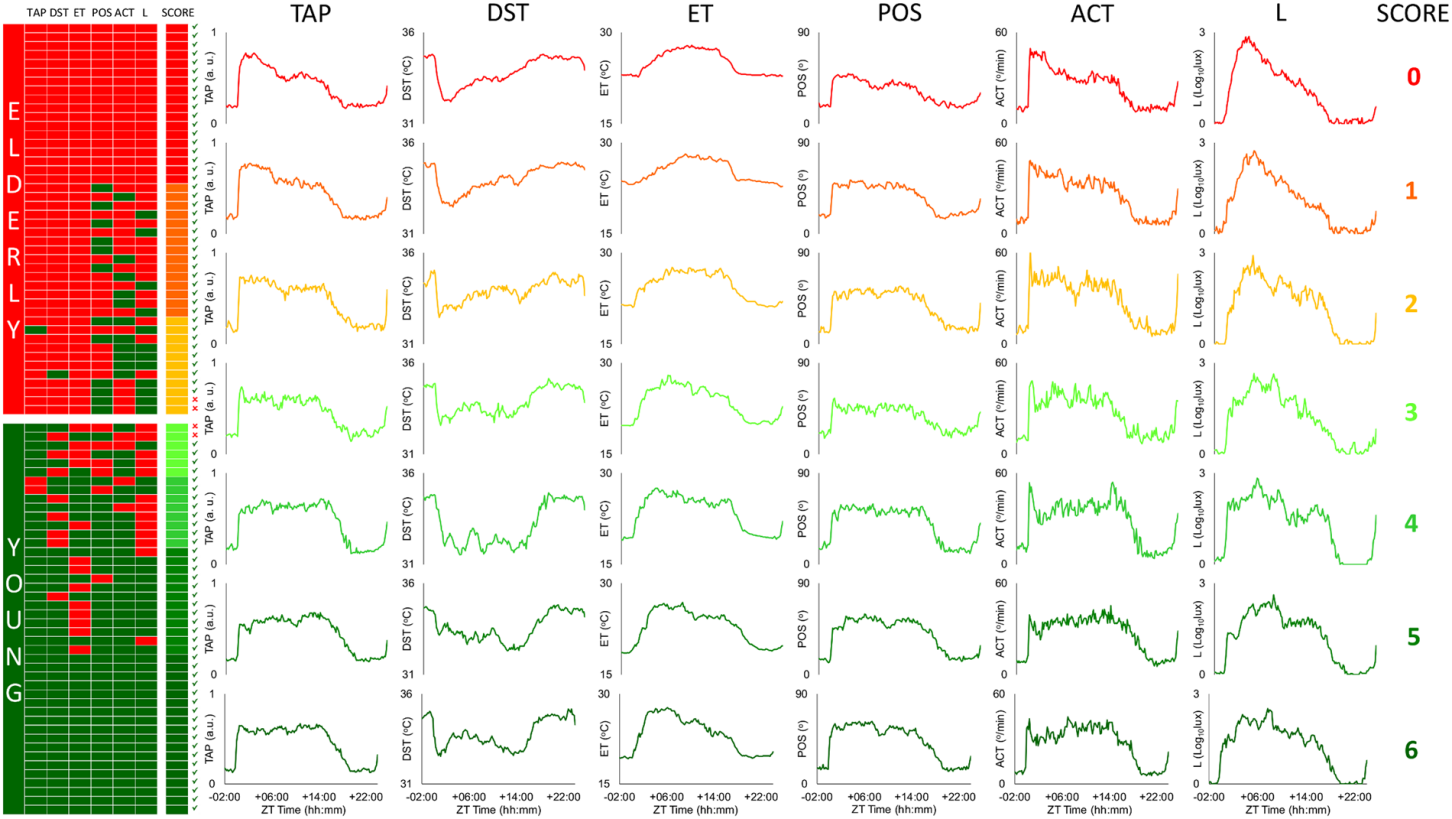
Macroarrays according to global score and agreement rates. Each row represents one participant, ordered by global score from top to bottom (from low to high scores, respectively). Rhythmic variables are ordered from left to right, according to their agreement rate for predicting age (TAP, DST, ET, POS, ACT and L). A red cell indicates that the subject was classified as aged, while a green cell indicates that the subject was classified as young. A global score column (again in a gradient from red to green), as well as a green checkmark (correct global classification) or a red cross (incorrect global classification), are shown following the variable columns. Next to the macroarrays, sleep offset standardized mean waveforms for each punctuation in the global score are represented and ordered from left to right, according to their agreement rate for predicting age and from top to bottom, as scores increased (as described above).

The global decision tree (Fig. 4A) attained an agreement rate of 95.3% (Sensitivity = 95.5%; Specificity = 95.2%), using a score of 3 as the cut-off point, i.e., a subject with a global score of 3 or more was classified as young, whereas a participant with a global score of 0, 1 or 2 was classified as elderly. It is worthy to note that the decision tree mismatches were located around the cut-off point (Fig. 5). Finally, the classification of the subjects according to this scoring and their respective agreement rates are shown in Fig. 4B.

Figure 5 shows the graphic matrix (*macroarrays*) for our population, divided by age group (red for aged and green for young participants). Each row represents a subject ordered according to his or her global score (located in the last column). Rhythmic variables are ordered from left to right, according to their agreement rate for predicting age. These columns are followed by the global scoring, where a green checkmark indicates a correct classification and a red cross an incorrect one. Standardized mean waveforms for sleep offset in each subscore of the global score are represented and ordered from left to right, according to their agreement rate for predicting age (as before) and from top to bottom, according to the global score (from low to high scores, respectively). It is worthy to note how all rhythmic variables change their pattern according to the global score, especially at the transition point from young to aged.

## Discussion

Our results show, for the very first time using this type of integrated analysis, that circadian rhythms can be used as an aging predictor. This prediction is based on the differences between young and aged individuals in terms of lifestyle habits, such as activity; synchronizers, such as light exposure; and endogenous variables, such as DST.

The DST rhythm showed a pattern similar to that previously described for these populations^22,26,27^. Thus, young participants had a marked wake maintenance zone in the evening^22,24,28^, both for arousal^29,30^ and DST^22^. Nevertheless, the wake maintenance zone in DST shifted to the morning in aged participants^22^, while arousal in the evening disappeared^30^. In addition, aged participants showed higher DST values during the evening than the young subjects, which is associated with sleepiness^21^ and could be caused by age-related thermoregulation impairments^19^.

Aged participants concentrated their maximum light exposure at noon, at which time it started to decrease progressively until reaching very dim light values in the evening^18,31,32^ and ending in a longer darkness period during sleep than has been previously reported^18,31^. Thus, the light exposure of aged participants seems insufficient for circadian synchronization, since low diurnal exposure is added to the normal aging of the circadian photoreception, which is impaired by lens yellowing^33^, cataracts^5^, reduction of ipRGCs in the retina and atrophy of ipRGCs dendritic trees^6^, all of which are important causes of chronodisruption^5^. In young participants, the peak occurred later and darkness during the night was shorter than in the elderly subjects, and during the late afternoon and evening they evidenced a plateau, probably due to indoors light exposure or electronic devices, as previously described^24,28,34,35^ and in accordance with a trend toward eveningness^36^. Thus, it would be important to consider specifically blue light exposure during the night in order to identify likely sources of this light exposure and implement the corresponding corrective countermeasures.

The environmental temperature pattern showed lower temperatures at night than during the daytime, as previously described^21,24,28^. However, the mean environmental temperature was warmer, phase delayed and had a lower day/night contrast in aged participants. The decrease in the capacity to perceive warm stimuli characteristic of aging^19^ would probably justify why these participants prefer to expose themselves to warmer environments. Ageing impairs cold-exposure autonomic response due to a decrease in the rate of discharge and the functionality of the sympathetic nerves^37,38^, and it also reduces parasympathetic vasodilation^39^. However, this preference for warmer environments at night could induce sleep disturbances, due to the increased difficulty to dissipate heat^40–42^.

Patterns of activity and body position showed low values during sleep and higher values during the active phase, as expected^24,43–46^. There was a trend in aged participants to perform their main activities during the morning, which translated into a phase advance^13,17,47^. Aged participants spent more time inactive and in lying or semirecumbent positions than young participants, which has been related to the aging process^13,17^. These changes are associated with a sedentary lifestyle and entail an increased health risk^48^.

The integrated variable TAP (based on the information provided by distal skin Temperature, motor Activity and body Position) showed a pattern with stable low values at night and variable high values during the daytime^46^. However, aged participants exhibited higher values at night than the younger subjects, suggesting superficial sleep^49^. In addition, the higher fragmentation in aged participants found here has been related to greater obesity, metabolic risk^50^ and mortality risk^51^. Finally, the aged subjects were phase advanced, as compared to the young participants, as has already been described with regard to the aging characteristics for other variables studied^47^.

In order to avoid any circannual effect on the studied rhythms, ambulatory circadian monitoring was performed in a short-time window (three weeks). But additionally, any possible bias in this sense, including sleep habits in different countries or communities was corrected by standardizing all phase markers by sleep offset as described by Kanterman *et al*.^52^. Thus, considering that all indexes introduced in the decision trees were standardized by sleep offset, they should be valid for any season and country or community.

The aforementioned differences between circadian rhythms in aged and young participants allow us, for the very first time using this type of integrated analysis, to differentiate between them without taking into account their biological age, but rather characteristics of their circadian patterns. Thus, lying positions during the daytime (MAX), a phase advance of light exposure during the rest period (TMIN standardized by SOFF), a phase advance of daytime environmental temperature (TM5 standardized by SOFF), activity (TMAX standardized by SOFF), distal skin temperature (TMIN standardized by SOFF) and TAP (TMAX standardized by SOFF) were the distinctive characteristic of aged participants, and the inverse for young subjects, with agreement rates higher than 75% in all cases. The best variable to predict the age group was the integrated variable TAP (92%), followed by distal skin temperature (87.5%), both of which are variables with a clear endogenous component^26,28,44,53^. Finally, when all the decision trees were considered together, the agreement rate reached 95.3%, with only 4 mistakes out of 88 predictions. Thus, these decision trees allow us to classify correctly young and elderly subjects. However, predict subjects’ age is not possible since a categorical but no continuous index has been obtained and such approach would require a high amount of subject in a wide age range.

The aging process of the circadian system is associated with lower day-night contrast, higher fragmentation and a phase advance^17,22,47^, which are related to SCR frailty^23^. These differences in circadian parameters were very significant among age groups and this is why they could be implemented in decision trees as accurate aging predictors. In fact, several circadian parameters have been reported as early biomarkers of Alzheimer’s disease and mild cognitive impairment^26,54,55^, supporting its potential application as biomarkers for neurodegenerative diseases screening.

The age-related changes associated with SCR could be generated by the aging process itself or by behavioral changes, as suggested by the fact that aged individuals spent more time indoors with low light intensities and warmer environmental temperatures and were less active and spent more time in lying positions during the active phase. This reveals that aged participants would be exposed to weak zeitgebers, mainly during the afternoon, when they are inactive and experience sleepiness, which could lead to worse sleep quality due to the loss of day-night contrast^24^. On the other hand, a healthy circadian system implies synchronization, phase-coupling or co-modulation of different rhythms at the right moment^56^. Thus, aged populations could benefit from chronoenhancement strategies centered on promoting brighter illumination and more activity in the afternoons. In this population, ambulatory circadian monitoring could constitute a useful tool to evaluate not only circadian system aging, but also successful strategies to “rejuvenate” the circadian system, which should be demonstrated by future studies.

## Materials and Methods

### Subjects

For the present study, 44 undergraduate student volunteers (20 men and 24 women, 19–25 years old), residents in Murcia, Spain (latitude 38°01′N), and 44 healthy elderly volunteers (22 men and 22 women, 67–75 years old), residents in Toledo, Spain (latitude 39°52′N), were recruited (see Table 4 for demographic characteristics). It is worthy to note that ageing process increases Body Mass Index (BMI), with almost half of the elderly population being overweighted (BMI 25–30). In elderly people overweight is related to the lowest mortality, while normal weight has an increased mortality risk^57^. Thus, elderly participants BMI was not matched with the young participants BMI since it would imply a bias by higher mortality risk.

**Table 4:** Subjects characteristics by age group (elderly participants, n = 44; young participants, n = 44).

	Young	Elderly
Men/Women	20/24	22/22
Age	19.14 ± 0.14	73.36 ± 0.56^‡^
Height	171.44 ± 0.01	164.97 ± 0.01^‡^
Weight	65.54 ± 1.98	77.54 ± 1.96^‡^
Body Mass Index	22.09 ± 0.53	28.49 ± 0.70^‡^

Values are expressed as the mean ± SEM. ^‡^p < 0.001, according to Welch t test.

Participants were instructed to wear all sensors for 5 consecutive weekdays, and were also encouraged to maintain their habitual lifestyle. All data were recorded under free-living conditions during the first two weeks of November. Participants were instructed to complete a sleep diary designed by the Chronobiology Laboratory at the University of Murcia, where they wrote down when sensors were removed.

The study abided by the bioethical principles set out in the Declaration of Helsinki and approved by the Ethical Committee of the University of Murcia in compliance with the Spanish RD53/2013 Law. Data from volunteers were protected according to Spanish Law 15/1999, of 13 September. All participants received appropriate information about the study characteristics and signed an informed consent form prior to their inclusion in the study.

### Distal skin temperature measurement

All subjects wore a Thermochron iButton DS1921H (Maxim Integrated Products, Sunnyvale, California, USA) with a precision of ±0.125 °C. This temperature sensor was positioned on the wrist of the non-dominant hand over the radial artery and isolated from the environmental temperature by means of a double-sided cotton sport wrist band, in order to measure DST as previously described^27^. The device was programmed to sample every 10 min over the course of 5 days.

### Body position and activity rhythm measurement

Body position and activity rhythms were assessed every 30 seconds, using a HOBO Pendant G Acceleration Data Logger UA-004-64 (Onset Computer, Bourne, Massachusetts, USA) placed on the non-dominant arm, with its X-axis parallel to the humerus bone. The manufacturing specifications and the method to obtain these variables have been previously described^46^. Activity is measured as the rate of change in degrees per minute, and position represents the tilt of the accelerometer X axis (parallel to the humerus bone of the arm), expressed in degrees (90° vertical arm position, 0° horizontal arm position). Both variables were averaged every 10 minutes to achieve a sampling frequency that matched that used for DST.

### Environmental temperature and light exposure recording

All subjects were also required to wear a HOBO Pendant Temperature/Light Data Logger UA-002-64 (Onset Computer, Bourne, Massachusetts, USA) on a neck chain to record environmental temperature and light exposure. Manufacturing specifications, memory, spectrum and accuracy for light exposure have been described in a previous work^35^. Light intensities in lux were converted into logarithmic units and averaged every 10 min to allow for comparisons with DST data^35^. For environmental temperature, the data logger had the following specifications: measurement range of between −20 and 70 °C, memory capacity of up to 28,000 values at regular preprogrammed intervals (in our case, every 30 seconds), accuracy of ±0.53 °C and resolution of 0.14 °C. Again, environmental temperatures in degrees Centigrade were averaged every 10 min to allow for comparisons with DST data.

### Calculation of the integrated variable TAP

Distal skin Temperature, Activity and body Position were normalized and averaged to obtain the TAP variable as already described (for more details, see Ortiz-Tudela *et al*.^46^). A TAP value of 0 indicates rest, a lying position and high distal skin temperature, which is compatible with sleep, while 1 indicates that the subject is standing, active and presents low distal skin temperature, which is consistent with a high arousal state (for more details, see Ortiz-Tudela *et al*.^26^).

### Data analysis

Artifacts produced by temporarily removing the sensors were filtered out of the raw data (leaving the datum empty). In addition, in order to eliminate atypical data, the interquartile distance (from Q1 to Q4) was calculated, and thus timepoints for which the rate of change with respect to the previous value was higher than the interquartile distance were eliminated (leaving the datum empty)^58^. The mean daily pattern for all variables was calculated per individual, and then averaged per group.

To analyze any possible differences between circadian patterns, we performed a calculation of non-parametrical indexes. The Circadian Function Index (measure of robustness, CFI), Interdaily Stability (constancy of the 24-h rhythmic pattern over days, IS), Intradaily Variability (rhythm fragmentation, IV) and Relative Amplitude (amplitude, RA) were calculated^46,59^. For variables with the acrophase during the daytime (activity, position, light exposure, environmental temperature and TAP), RA was calculated as the difference between M10 (average for the 10 consecutive hours with the maximum values, measured in 10-min intervals) and L5 (average for the 5 consecutive hours with the minimum values, measured in 10-min intervals), divided by the sum of M10 and L5, as previously reported by Witting *et al*. (1990) for activity^59^. However, since the DST acrophase occurs during the rest period, RA was calculated as the difference between M5 (average for the 5 consecutive hours with the maximum values, measured in 10-min intervals) and L10 (average for the 10 consecutive hours with the minimum values, measured in 10-min intervals), divided by their sum^35^. Thus, minimum values (L10 for DST and L5 for the remaining variables) were denoted as MIN, while maximum values (M5 for distal skin temperature and M10 for the other variables) as MAX, and their corresponding timing as TMIN and TMAX. In addition, regarding light exposure, we calculated time spent during the morning (08:00 to 15:50 h), evening (16:00 to 23:50 h) or at night (00:00 to 07:50 h) at different light intensities (<10 lux, 10–100 lux, 100–1000 lux, 1000–10000 lux and >10000 lux) for each subject.

In order to standardize mean daily patterns and phase variables (TMIN and TMAX) obtained from the non-parametrical indexes, making them useful for other countries with different schedules or official times, the phase variables were relativized to sleep offset (SOFF) and named as TMIN-OFF and TMAX-OFF. Sleep offset was determined from the sleep logs.

For the classification of the subjects as aged or young people, the aforementioned non-parametrical indexes for DST, activity, position, light exposure, environmental temperature and TAP were entered into WEKA version 3.8.1 (University of Waikato, Hamilton, New Zealand)^60^. An independent classification was then performed for each variable (DST, activity, position, light exposure, environmental temperature and TAP) by means of a J.4.8 decision tree that uses the C 4.5 algorithm for decision making^61^, which selects the decision that maximizes the information gain at each step. In order to simplify the algorithm, all decision trees were required to use just one decision. Each individual decision tree corresponds to the best of 100 iterations performed with 66% of the data randomly selected and checked against the other 33%. Then, specificity (test’s ability to correctly detect elderly subjects), sensitivity (test’s ability to correctly detect young subjects) and agreement rate for every individual decision tree were calculated. Thus, six independent decision trees were developed (one per variable), which were then combined into a unique algorithm (global decision tree). This combination was performed as follows: every time that a decision tree defined a pattern as young, it was given a score of 1, and otherwise, it was assigned a score of 0. As a result, each subject had a score between 0 (every decision tree defines him/her as an elderly person) and 6 (when defined as a young person in all cases).

All data are expressed as the mean ± SEM and were processed using Microsoft Office Excel 2016. Statistical comparisons between young and elderly people were conducted by means of Welch t test for demographic data. For exposure time at different light intensities and circadian non-parametrical indexes analysis a general linear model controlled by gender with *post-hoc* Benjamini-Hochberg procedure for multiple comparisons was performed. All statistical analyses were conducted with SPSS version 23.0 (SPSS, Inc. Chicago, IL, USA).

## Electronic supplementary material

Supplementary information
