# Supplementary material for: Circadian monitoring as an aging predictor
Source: Sci Rep. 2018 Oct 9;8:15027. doi: 10.1038/s41598-018-33195-3 (PMC6177481; doi:10.1038/s41598-018-33195-3)
Supplement: Supplementary file 1 — Supplementary information [file 41598_2018_33195_MOESM1_ESM.docx]

**Circadian monitoring as an aging predictor**

Martinez-Nicolas A^1,2^, Madrid JA^1,2^, García FJ^2,3^, Campos M^4^, Moreno-Casbas MT^2,5^, Almaida-Pagán PF^1,2^, Lucas-Sánchez A^1,2^, Rol MA^1,2^

^1^Chronobiology Lab, Department of Physiology, College of Biology, University of Murcia, Mare Nostrum Campus. IUIE, IMIB-Arrixaca, Spain.

^2^Ciber Fragilidad y Envejecimiento Saludable (CIBERFES), Madrid, Spain.

^3^Geriatrics Section, Hospital Virgen del Valle, Toledo, Spain

^4^Department of Computer Science and Systems, University of Murcia, IMIB-Arrixaca, Murcia 30100, Spain

^5^Nursing and Healthcare Research Unit (Investén-isciii), Madrid, Spain

Corresponding author:

Rol MA, [angerol@um.es](mailto:angerol@um.es).

**SUPPLEMENTARY TABLE 1. Non-parametrical analysis of circadian rhythm phase standardized by sleep offset according to age group.**

|  | | **Young** | **Elderly** |
| --- | --- | --- | --- |
| DST | TMIN – OFF | 08:40 ± 00:40 | 05:11 ± 00:16^‡^ |
|  | TMAX – OFF | 19:04 ± 00:35 | 18:39 ± 00:27 |
| L | TMIN – OFF | 20:40 ± 00:08 | 19:25 ± 00:19^‡^ |
|  | TMAX – OFF | 07:37 ± 00:21 | 06:16 ± 00:18^‡^ |
| ET | TL10 – OFF | 20:29 ± 00:15 | 19:55 ± 00:37 |
|  | TMIN – OFF | 21:23 ± 00:15 | 21:40 ± 00:31 |
|  | TMAX – OFF | 07:45 ± 00:23 | 08:47 ± 00:36 |
|  | TM5 – OFF | 06:43 ± 00:32 | 08:34 ± 00:44^‡^ |
| ACT | TMIN – OFF | 20:26 ± 00:08 | 19:23 ± 00:16^‡^ |
|  | TMAX – OFF | 09:37 ± 00:20 | 06:40 ± 00:22^‡^ |
| POS | TMIN – OFF | 20:29 ± 00:11 | 19:01 ± 00:38^#^ |
|  | TMAX – OFF | 07:37 ± 00:17 | 06:36 ± 00:34^#^ |
| TAP | TMIN – OFF | 20:30 ± 00:08 | 19:16 ± 00:14^‡^ |
|  | TMAX – OFF | 09:23 ± 00:18 | 05:48 ± 00:14^‡^ |

Timing of the 5 consecutive hours with the highest values, standardized according to TMAX-OFF and the 10 consecutive hours with the lowest values, standardized according to TMIN-OFF for distal skin temperature. For light exposure, environmental temperature, activity, body position and the integrated variable TAP, the 10 consecutive hours with the highest values, standardized according to TMAX-OFF and the 5 consecutive hours with the lowest values, standardized according to TMIN-OFF. Values are expressed as mean ± SEM. ‡p<0.001 and #p<0.01, according to the General Linear Model.
